# The influence of rotational error and axial shift of toric intraocular lenses on residual astigmatism

**DOI:** 10.1371/journal.pone.0311566

**Published:** 2024-12-05

**Authors:** Diana Gargallo, Laura Remón, Jorge Ares, Francisco J. Castro-Alonso

**Affiliations:** 1 Department of Applied Physics, University of Zaragoza, Zaragoza, Spain; 2 UFR, Department of Ophthalmology, University Hospital Miguel Servet, Zaragoza, Spain; 3 GIMSO, Institute for Health Research Aragón, Hospital Universitario Miguel Servet, Zaragoza, Spain; Saarland University, GERMANY

## Abstract

**Purpose:**

Accurate alignment of Toric Intraocular Lens (T-IOLs) in cataract surgery is crucial for good visual outcomes. The purpose of this study was to evaluate the influence of rotation, axial shift and their combined effects on the refractive error and image quality of a wide range of T-IOL powers (from +1.50 D to +6.00 D cylinder) and two pupil diameters (3.34 and 4.44 mm).

**Methods:**

Numerical ray tracing was utilized to quantify the residual error. Simulated retinal images and Visual Strehl (VS) ratios were calculated to evaluate image quality.

**Results:**

First, T-IOL rotation showed better agreement with Holladay’s formula than 3.33% rule. Second, axial displacement resulted in acceptable residual cylinder (<0.50 D) across all examined cylinder powers. Third, concerning the combined effects, the influence of axial shift on residual cylinder becomes negligible when rotation errors exceed 2.5°. Fourth, a pupil-dependent nonlinear relationship was noted for image quality caused by both types of misalignment factors.

**Conclusions:**

The 3.33% rule was confirmed as a reasonable approximation for the residual astigmatism caused by rotation of T-IOLs. The influence of axial shift on residual astigmatism becomes insignificant when there is also rotation. Image quality studies confirm that 30° of rotation are enough invalidate the compensation benefits of a T-IOLs in comparison with a Spherical Intraocular lens.

## Introduction

Toric Intraocular Lenses (T-IOLs) have gained popularity as effective choices for compensating corneal astigmatism during cataract surgery. Prior to the development of T-IOLs, surgeons often relied on corneal relaxing incisions to correct astigmatism [1]. This technique presented limitations including unpredictable healing, inaccurate positioning of incisions and corneal high-order wavefront aberrations [2, 3]. Multiple studies have demonstrated that the implantation of T-IOLs is a safe and effective method for treating corneal astigmatism in a single surgical procedure [4–6]. The success of T-IOLs is not possible without taking care of some key points [7, 8]: 1) knowledge of the pre-existing corneal astigmatism [8, 9]; 2) predictability of the surgically induced astigmatism [8]; 3) correct alignment of the IOL [8, 10–12]; 4) predictability of the T-IOL axial position [13, 14]; and 5) available range of cylinder powers from manufacturers [15]. Rotational [12] and axial positioning errors of the T-IOL [16] are the most important factors to generate post-surgery refractive error. Once the T-IOL is implanted, axial and rotational stability depend on improper fixation of the crystalline lens, capsular bag contraction, zonular fiber rupture and secondary cataract [17]. Several clinical studies [18, 19] found a positive correlation between IOL rotation and axial length. Shah *et al*. [18] reported that the rotation of a T-IOL was more common in highly myopic eyes. Similar results were found by Zhu *et al*. [19].

The first clinical study collecting the postoperative refractive results after T-IOL implantation was conducted by Shimizu *et al*. [20] Eyes with T-IOL rotations over 30° showed postoperative astigmatism that exceeded the targeted correction. Since then, it has been recognized that an accurate alignment of T-IOL is crucial to achieve the intended reduction of astigmatism at the time of cataract surgery. Several theoretical studies based on paraxial approximation predicted the residual error when T-IOLs rotate. Felipe *et al*. [21] concluded that T-IOL rotations less than 10° give satisfactory astigmatism correction. Langenbucher *et al*. [22] found that a 1-degree T-IOL misalignment resulted in a residual cylinder of 3.33% of the initial power. Holladay *et al*. [23] found that with a rotation above 30°, the residual astigmatism exceeds the original astigmatism. The clinical study by Shimizu *et al*. [20] supported the predictions of paraxial theoretical studies.

Probably motivated by these studies, a clinical guideline known as the “3.33% rule” was established to predict the relationship between residual cylinder and rotation error of the T-IOL. The 3.33% rule is that the Residual Cylinder (Resid_CYL) resulting from a rotation error in degrees (Alpha) maintains a linear relationship with the cylinder (CYL) intended to be compensated by a toric lens as follows: Resid_CYL = CYL×Alpha×3.33/100. According to this rule, the residual cylinder exceeds the intended astigmatism when the lens rotates more than 30°.

Subsequent research has challenged this rule, proposing a more complicated relationship [24–26]. Tognetto *et al*. [27] conducted an experimental evaluation of image quality as a function of rotation error. Image quality was analyzed using the Visual Information Fidelity (VIF) index [28]. A nonlinear relationship was found between the VIF and T-IOL rotation. At 45° rotation of the T-IOL, the VIF was the same as that for a “no toric correction”. In this study, all images were obtained with a 3 mm pupil diameter and one T-IOL (+3.75 D cylinder and +21.00 D of Spherical Equivalent (SEQ)).

To our knowledge, the impacts of T-IOL rotation, axial shift and their combined effects have not been quantified without the paraxial approximation formalism. The aim of this study is to investigate the impact of this T-IOLs misalignment on residual error and on visual quality. The process involved a numerical ray tracing analysis with the creation of a 16 pseudophakic eye models.

## Methods

Sixteen geometric pseudophakic eye models were implemented and analyzed using a commercial optical design software (OSLO EDU Edition 2001–2012, Revision 6.6.0 –Lambda Research Corporation). For practicality, although in a manner opposite to what happens in clinical practice, corneal biconical models were tailored to achieve complete astigmatic compensation in combination with a representative T-IOL set.

### Toric intraocular lens design

Sixteen T-IOLs were designed by combining four SEQ values [+16.00, +20.00, +24.00 and +28.00 D] with four cylinder values [+1.50, +3.00, +4.50 and +6.00 D]. The cylinder powers corrected with-the-rule corneal astigmatism at the corneal plane of +1.08, +2.18, +3.27 and +4.32 D, respectively. T-IOLs had refractive index n = 1.554 at wavelength λ = 555 nm. The toric surfaces of the IOLs were on the front of the lens. The posterior surfaces of all lenses were spherical with a power of 10.9D. Edge thickness was 0.210 mm for an optical diameter of 6.00 mm.

### Eye models

Sixteen geometric pseudophakic eye models with with-the-rule anterior corneal astigmatism and two different entrance pupil diameters (3.34 mm and 4.44 mm) were created. The front surface of the corneas was biconical, with a horizontal X meridian based on data from the Atchison’s eye model [29] and the vertical Y meridian adjusted for each model of T-IOL to present the opposite astigmatism to the IOL (see Table 1). The IOL position was set following the research conducted by Castro *et al*. [30] (Table 2). A constant axial distance of 2.72 mm from the posterior surface of the cornea to the iris plane was kept across all eye models.

**Table 1:** Eye model used for T-IOL designs and simulations.

Surface	Radius (mm)	Conic Constant	Type of surface	Thickness (mm)	Refractive index at 555 nm
**Anterior Cornea**	X meridian: 7.77 Y meridian: was determined according to the design of the T-IOL	-0.150	Biconic	0.550	1.3760
**Posterior Cornea**	6.40	-0.275	Prolate Ellipse	3.150	1.3374
**Stop**	Infinite	-	-	* See Table 2	1.3374
**IOL Anterior Surface**	Different for each T-IOL	0	Toric	0.633	1.554
**IOL Posterior Surface**	-20.00	0	Spherical	As required to minimum RMS wavefront error	1.3360
**Retina**	-12.00	0	Spherical		

**Table 2:** Axial position of the T-IOLs according to Castro *et al*. [30].

SEQ (D)	16				20				24				28			
CYL (D)	1.50	3.00	4.50	6.00	1.50	3.00	4.50	6.00	1.50	3.00	4.50	6.00	1.50	3.00	4.50	6.00
**Stop to IOL Anterior Surface (mm)**	1.58	1.58	1.58	1.58	1.20	1.20	1.20	1.20	1.20	1.20	1.20	1.20	0.88	0.88	1.20	1.20

To achieve emmetropia, the vitreous chamber depth was set to minimize the Root Mean Square (RMS) wavefront error for the smaller entrance pupil diameter. The eye models, comprising the cornea and T-IOL, exhibited a fourth-order Zernike standard American National Standard Institute (ANSI) Spherical Aberration (SA) of +0.568, +0.588, +0.603 and +0.624 μm for +16.00, +20.00, +24.00 and +28.00 D spherical equivalents, respectively, with a +3.00 D cylinder and a 6.00 mm entrance pupil diameter. SA values were practically independent of the cylinder value of the lenses with a maximum observed difference between SEQ28_CYL1.50 and SEQ28_CYL6.00 of 0.061 μm.

### Numerical simulations

**Fig 1:**
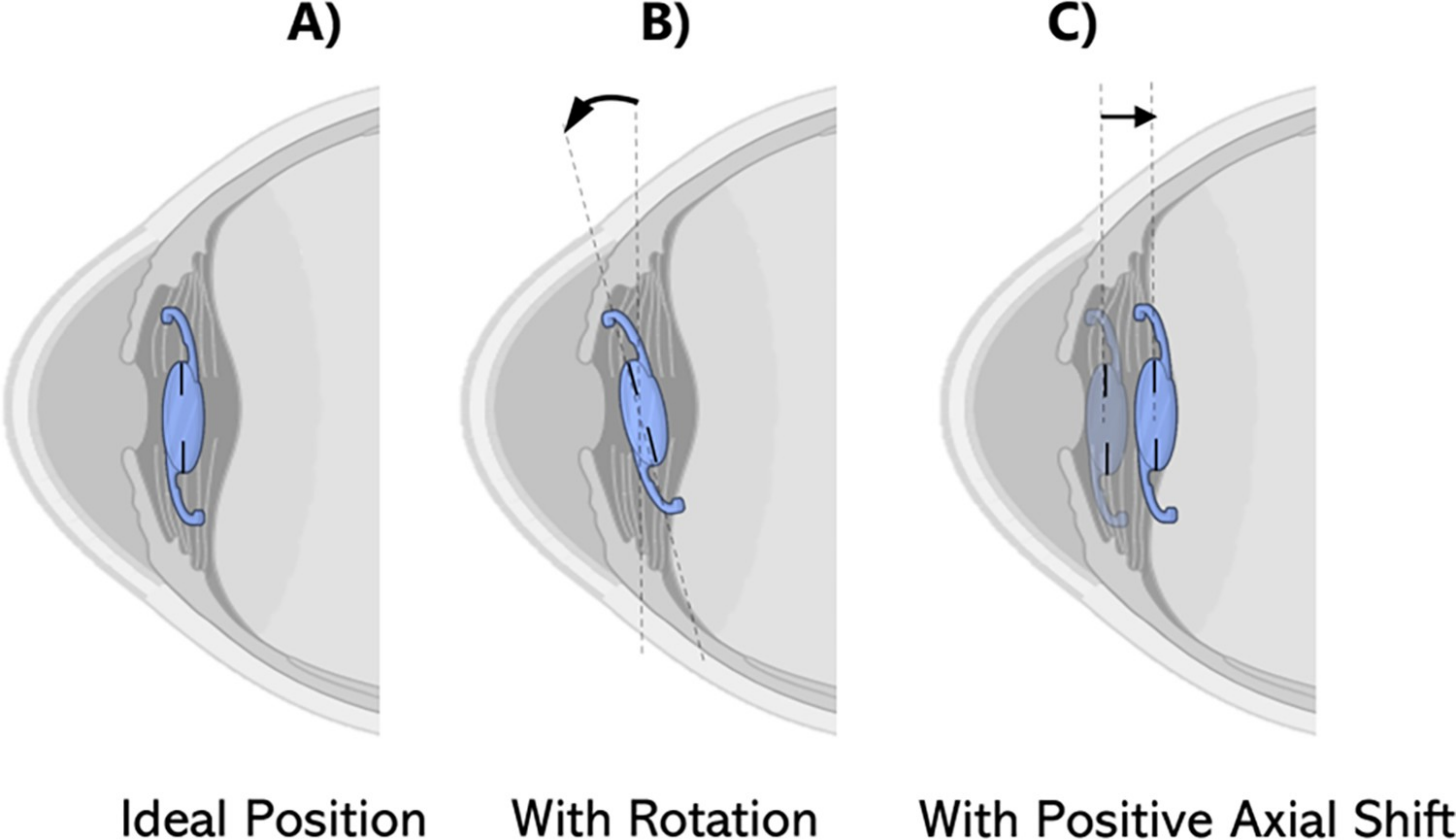
A) Ideal position, B) T-IOL with rotation and C) with positive axial shift.

An assessment of T-IOL optical performance was performed using OSLO Edu for different alignment conditions. First, the case in which the lens is correctly aligned (Fig 1A) was considered. Subsequently, the T-IOLs (Fig 1B) underwent a rotational misalignment, starting from the correct position in 2.5° increments up to 10°, followed by 5° increments up to 30°. Rotation was carried out both clockwise (positive) and counterclockwise (negative), as viewed from the front of the eye.

Axial shifts were also applied to the T-IOL (Fig 1C). This involved axial displacements of ±0.10, ±0.25, ±0.50, ±0.75 and ±1.00 mm and relative to the ideal position. Positive values indicate T-IOLs displacements towards the back of the eye.

For each rotation, axial shift and their combinations, numerical ray tracing from an infinitely far object point was performed to estimate the residual wavefront aberration expressed as a polynomial Zernike sum. The obtained Zernike coefficients were used to estimate [31] the refraction at the Entrance Pupil (EP) plane. With this methodology, it is being assumed that the wavefront error obtained from an ingoing ray tracing starting from a far point closely approximates that which can be obtained from an outgoing ray tracing starting at the retina [32].

Two different paraxial entrance pupil diameters of 3.34 mm and 4.44 mm were evaluated. Pupil diameters respectively correspond to 3.00 mm and 4.00 mm aperture stop diameters when pupil aberrations are neglected. The following equations were applied to obtain the diopter vector components [33] of residual refractive error that achieve minimum RMS wavefront error at the entrance’s pupil plane:

$$M_{\_EP}=\frac{-4\sqrt{3}C_{2}^{0}}{R^{2}}$$

$$J_{0\_EP}=\frac{-2\sqrt{6}C_{2}^{2}}{R^{2}}$$ (1)

$$J_{45\_EP}=\frac{-2\sqrt{6}C_{2}^{-2}}{R^{2}}$$

where *M*__*EP*_ denotes the average spherical error, *J*_0_*EP*_ and *J*_45_*EP*_ indicate the components of astigmatism and oblique astigmatism respectively, $C_{2}^{0}$ is the Zernike defocus coefficient, $C_{2}^{2}$ is the Zernike astigmatism and $C_{2}^{-2}$ is the Zernike oblique astigmatism coefficient (all in μm), and R is aperture radius (in mm) of the entrance pupil’s system [34].

The residual sphero-cylindrical refraction in minus cylinder form was obtained with the following equations:

$${Cyl}_{EP}=-2\sqrt{{J_{0\_EP}}^{2}+{J_{45\_EP}}^{2}}$$

$${sph}_{EP}=M_{\_EP}-\frac{{Cyl}_{EP}}{2}$$ (2)

$$\alpha_{EP}=\frac{1}{2}{tan}^{-1}(J_{45\_EP}{/J}_{0\_EP})$$

where *sph*_*EP*_ and *Cyl*_*EP*_ are the spherical and cylindrical components respectively, of the residual sphero-cylindrical correction at the entrance pupil plane, and *α*_*EP*_ is the axis orientation of the minus cylinder correction.

Considering the entrance pupil distance in relation to the corneal vertex plane (*d*_*EP*_), the residual sphero-cylindrical refraction at the corneal plane was determined as follows:

$$sph=\frac{{sph}_{EP}}{1+d_{EP}{sph}_{EP}}$$

$$Cyl=\frac{{{sph}_{EP}+Cyl}_{EP}}{1+d_{EP}({{sph}_{EP}+Cyl}_{EP})}-\frac{{sph}_{EP}}{1+d_{EP}{sph}_{EP}}$$ (3)

$$\alpha =\alpha_{EP}$$

where *sph*, *Cyl* and *α* are the residual sphero-cylindrical in minus cylinder form at the corneal plane. In these equations, *d*_*EP*_ must be specified in meters. Finally, the percentage effective loss of the T-IOL was also calculated as the residual cylinder divided by the preoperative corneal cylinder multiplied by 100. The viability of the calculated sphero-cylindrical refractions calculated was verified for the most extreme residual refraction errors.

### Simulated retinal images and Visual Strehl ratio

For each of the conditions, the Zernike coefficients of the wavefront aberration were obtained with OSLO Edu. Then, a standard Fourier filtering technique [35] were used for retinal image simulation of +0.20 logMAR optotypes. Image simulation using the best spherical correction in terms of minimum RMS aberration were also calculated. The image resulting from this simulation was named the “no toric correction”.

To provide an objective quality assessment, the Visual Strehl (VS) ratio was calculated. The VS ratio is a visual image quality metric with strong correlation to visual acuity and which is effective in predicting subjective best focus [36].

## Results

### Influence of the T-IOL rotation on residual refraction

**Fig 2:**
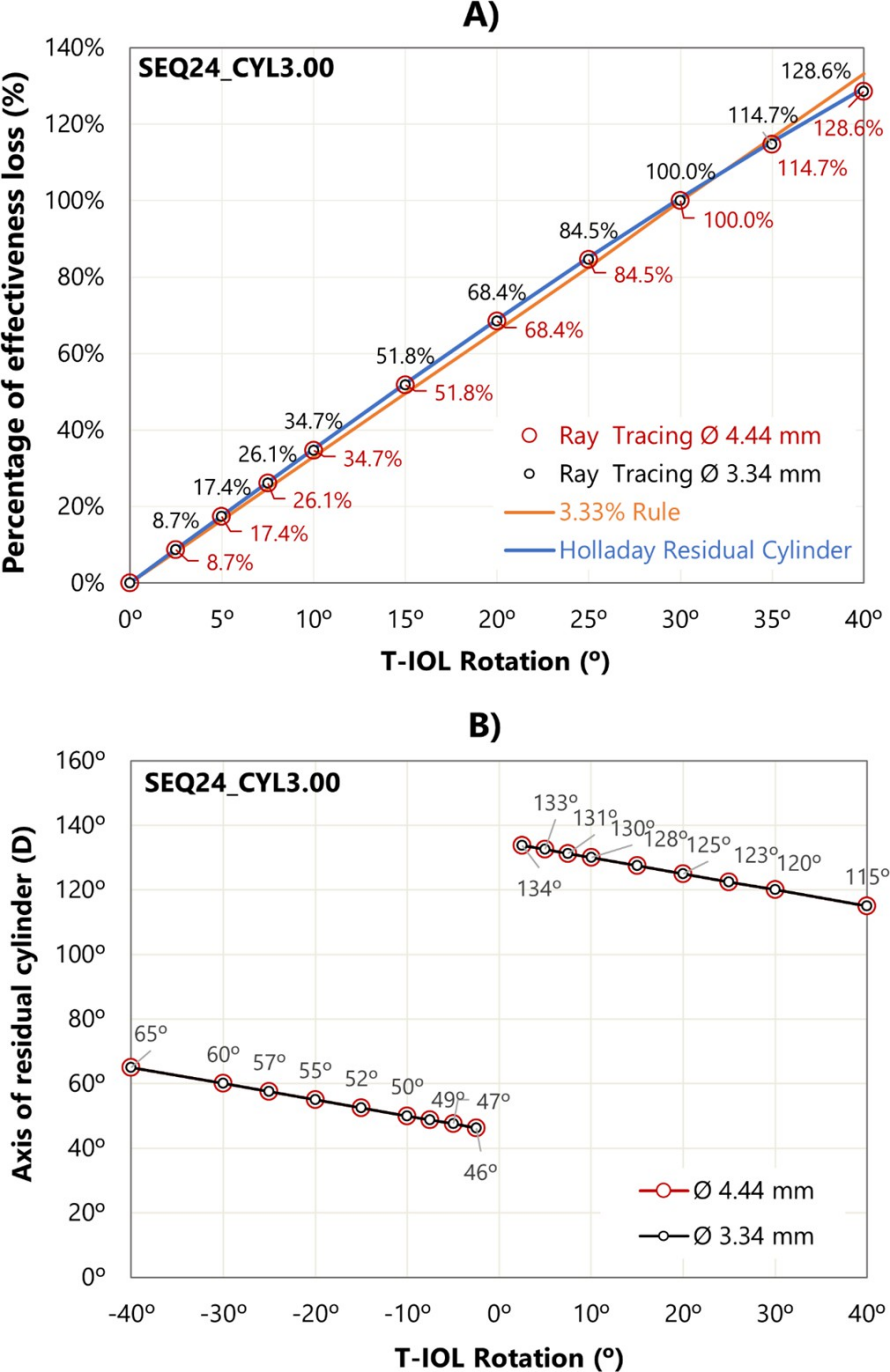
A) Percentage of effectiveness loss obtained by the three methods. B) Axis degrees for a minus cylinder correction as a function of the T-IOL rotation. These results are specific to the SEQ24_CYL3.00 eye model. Rotation was carried out for both clockwise (positive) and counterclockwise (negative).

Taking the SEQ24_CYL3.00 eye model as a representative case [37], Fig 2A shows the effective loss with rotation for two entrance pupil diameters. Numerical ray tracing results are compared with the “3.33% rule” and with the formula described by Holladay [38].

The three methods indicated zero cylindrical correction for a 30° rotation. Evaluating the data obtained from 0° to 30°, a mean difference of 1.70% was observed between the values obtained using the “3.33% rule” and this study, with the maximum discrepancy of 2.4% at 20° rotation. The differences between Holladay’s theoretical formula and numerical ray tracing were smaller, with an average difference of 0.4% from 0° to 30°, and a maximum discrepancy of 0.8% at 30° rotation. In general, the values derived from the “3.33% rule” were lower than those obtained using the other methods.

Fig 2B illustrates the residual cylinder axis for the SEQ24_CYL3.00 model eye when the T-IOL is rotated. Consistent with previous observations, nearly identical behaviors were noted for both pupil sizes.

**Fig 3:**
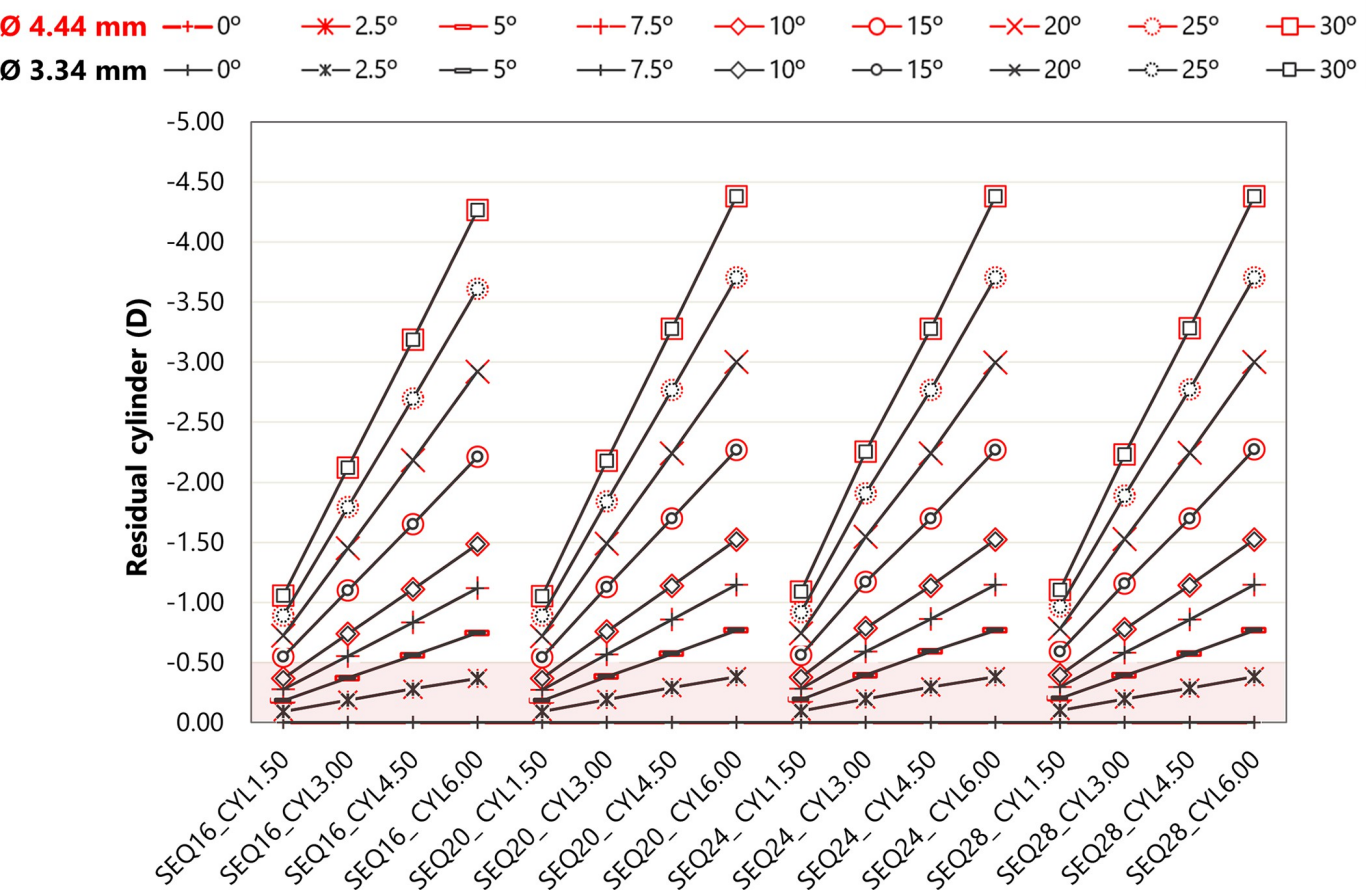
Residual cylinder at corneal plane as a function of T-IOL rotation for each eye model and both pupil diameters. The red zone indicates the clinically relevant level of residual cylinder (<0.50 D) [39].

Fig 3 shows the effect of T-IOL rotation on residual cylinder for each eye model and T-IOL. As expected, the rotation at which residual cylinder reaches a clinically relevant value of 0.50 D [39] depends on the cylindrical power. For example, a T-IOL with +1.50 D cylinder has a limit of 15°, whereas a T-IOL with a +6.00 D cylinder power has a limit of 2.50°. However, the SEQ power of the IOL has a minimal influence on the value of residual astigmatism caused by T-IOL rotation (the maximum difference was 0.11 D between SEQ16 and SEQ28 for a 30° rotation). The M component of the residual error was zero regardless of rotation error. The cylinder error caused by rotation was similar for both pupil diameters (Figs 2 and 3).

### Influence of the T-IOL axial shift on residual refraction

**Fig 4:**
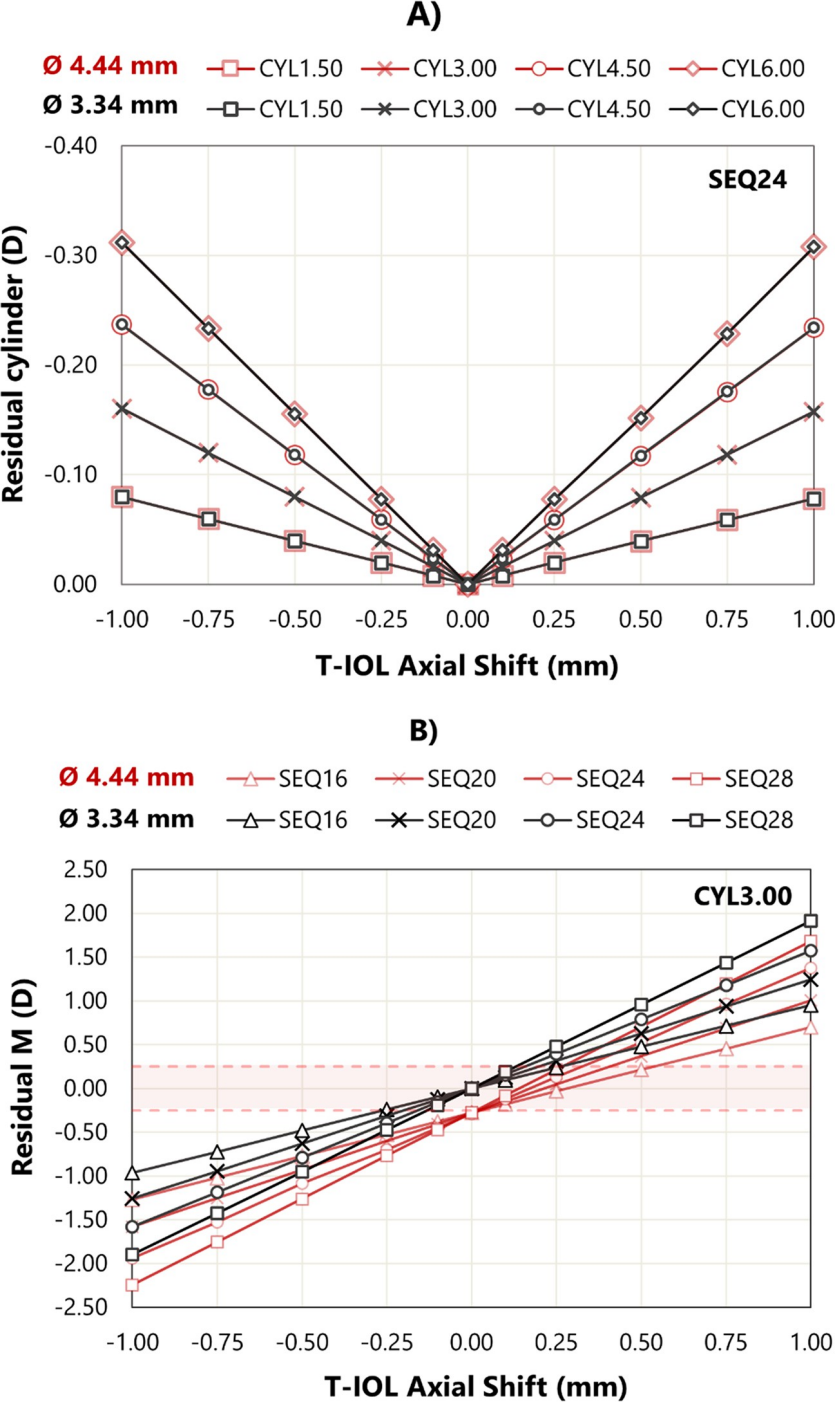
A) Residual cylinder at corneal plane as a function of axial shift for a SEQ24 and the four T-IOL cylinder values. B) Residual M as a function of axial shift for a CYL +3.00 D and the four SEQ values. Results are presented for two pupil diameters. The red zone indicates the clinically relevant level of residual cylinder <0.50D [39]. The value 0.00 on the X-axis represents the position of the T-IOL where emmetropia is achieved. Negative values represent positions in front of the position to achieve the emmetropia.

Fig 4A shows residual cylinder for the SEQ24 eye model and four T-IOL cylinder powers as a function of axial shift. For all axial shifts, the residual cylinder remains below the clinical limit of 0.50 D [39], nearly symmetrical with axial shift sign and independent of pupil diameter. For other, a maximum difference range of values of 0.008 D was found in across model eye.

Fig 4B shows the residual M values for the four SEQs and a CYL +3.00 D as a function of axial shift for both pupil sizes. For both entrance pupil diameters, the residual Ms are linear with the axial shift SEQ value. The slopes of the dependencies (D/mm) for an entrance pupil diameter of 3.34 mm are 0.958, 1.254, 1.577, and 1.908 D/mm for SEQ values of +16.00, +20.00, +24.00, and +28.00 D, respectively, and for a diameter of 4.44 mm, they are 0.985, 1.294, 1.654, and 1.963 D/mm, respectively.

For the larger pupil diameter, minimum residual M did not occur at the zero axial shift position. This finding can be explained considering that the eye model was optimized to minimize RMS wavefront aberration for the smaller pupil size. Because of the minimum change of SA with the cylinder power at our model eyes, similar behaviour was found for the other CYL values.

### Influence of combined T-IOL rotation and axial shift on residual refraction and axis

**Fig 5:**
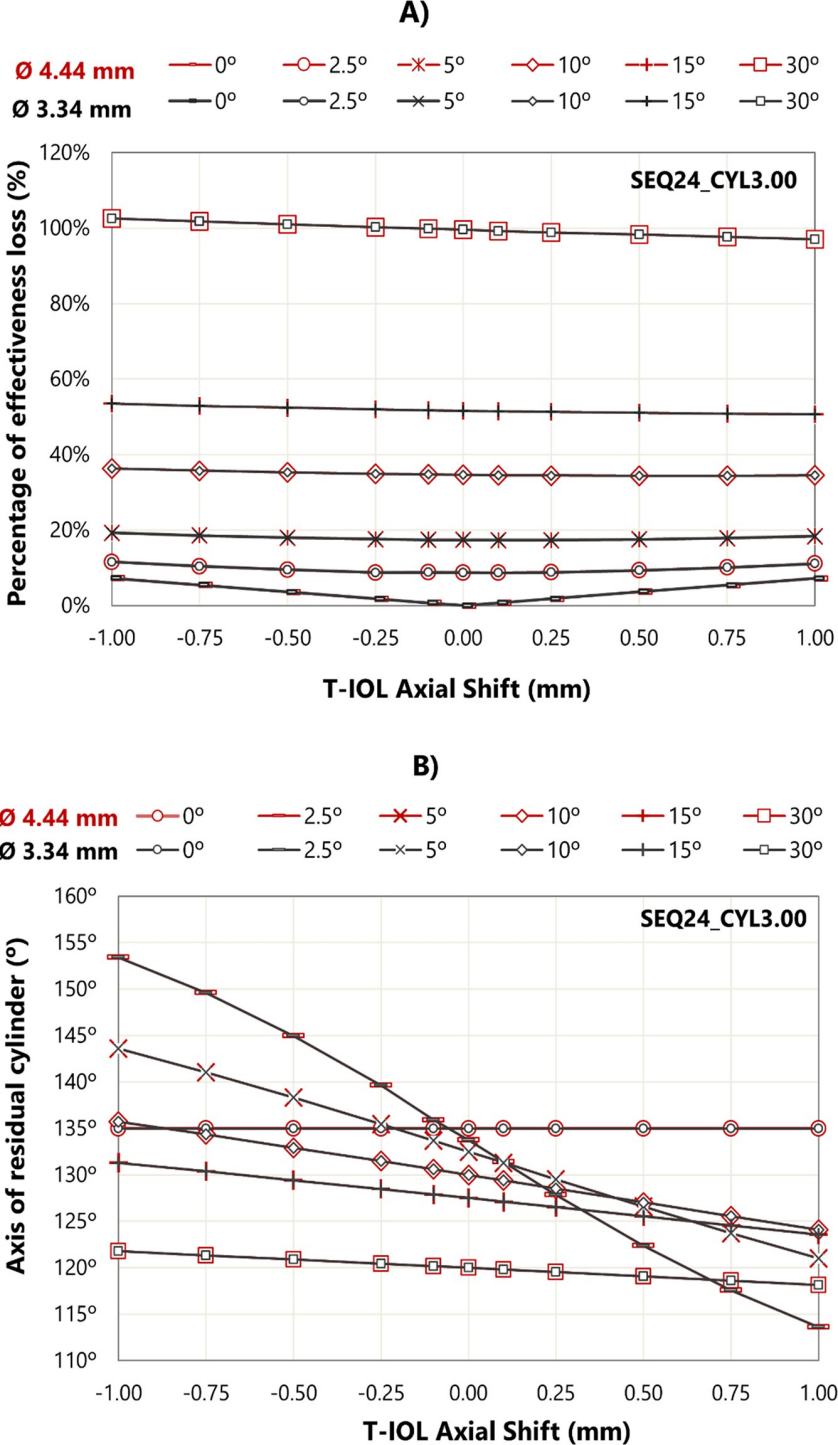
A) Residual cylinder as a percentage of preoperative magnitude resulting from axial shift and rotation B) Axis of residual cylinder due to axial rotation and axial shift. These results are for the SEQ24_CYL3.00 eye model.

Fig 5A shows the effective loss resulting from rotation and axial shift for the models with SEQ24. Similar results were observed in all other eye models, with a maximum difference in the residual cylinder of 1.56%.

Axial shift produces a small effective loss (> 7%) when the T-IOL is not rotated. Once rotation occurs, axial shift becomes inconsequential, and the effective loss variations at different axial shifts under the same rotation remaining less than 2%. For the larger rotation angles (15° and 30°), effective loss increases slightly more as the lens moves closer to the cornea than when it moves away.

Fig 5B shows the effects of combined rotation and axial displacement on the residual cylinder axis for T-IOL models with CYL3.00 and for both entrance pupil diameters. The results were almost independent of the T-IOL’s SEQ and cylinder, with a maximum variation of 1.05°.

If the T-IOL rotates 2.50°, the residual cylinder axis is 133°. If in addition to this rotation, the T-IOL is posteriorly displaced by 0.25 mm, the residual cylinder axis becomes 128°. This example demonstrates how the combination of rotation and axial displacement of the intraocular lens can influence the orientation of the cylinder correction in an eye with-the-rule anterior corneal astigmatism. As rotation increases, the influence of the T-IOL’s axial position on the residual cylinder axis decreases.

### Influence of the T-IOL rotation on image simulation

**Fig 6:**
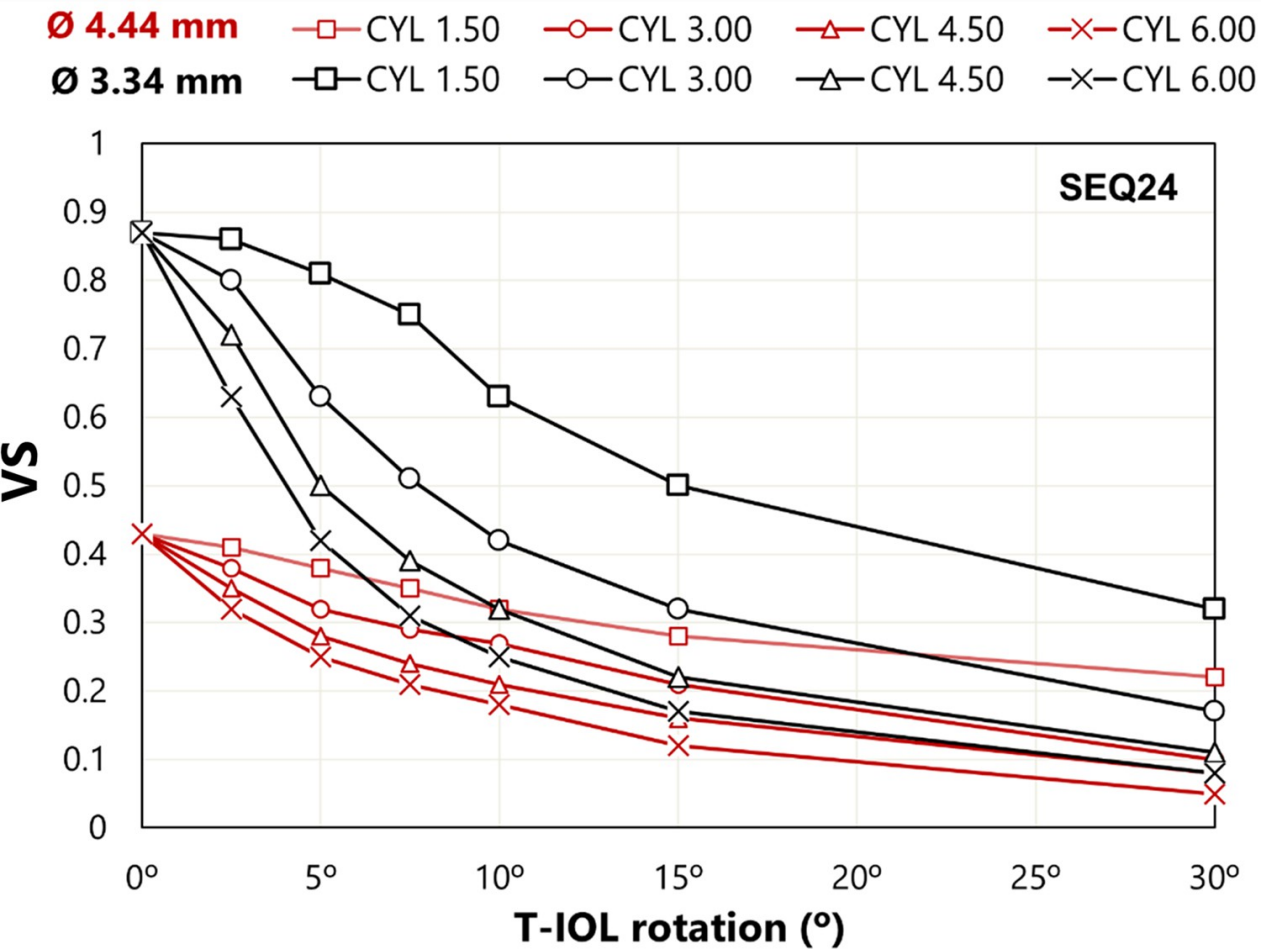
VS as a function of rotation for a SEQ24 with four values of CYL.

Fig 6 shows the VS ratio as a function of T-IOL rotation for a SEQ24 with four different cylinder values. As cylinder increases, there is a greater decline in visual quality with rotation. For example, with an entrance pupil diameter of a 3.34 mm, a T-IOL with a +1.50 D cylinder maintains a VS greater than 0.8 for rotations up to 5*°*, while a T-IOL with a +3.00 D cylinder does not maintain a VS above 0.8 beyond 2.50*°* rotation.

**Fig 7:**
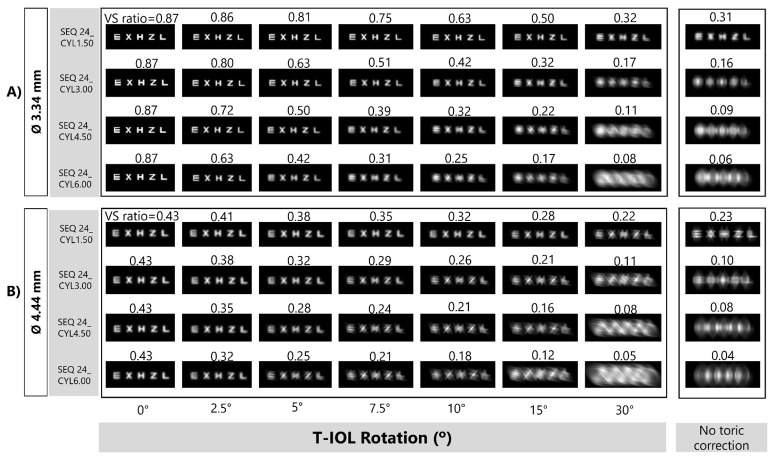
Simulated retinal images as a function of rotation for a SEQ24 with four values of CYL and different entrance pupil diameters: A) 3.34 mm and B) 4.44 mm. The “no toric correction” is shown for each cylinder in the last column. The VS value of each simulated image is shown at the top of the image. The line of letters represents 0.20 logMAR visual acuity.

Fig 7 shows simulation images for a SEQ24 and four cylinders and two pupil sizes, which support the results presented in Fig 6. For comparison, the image simulations with “no toric correction” are also presented. The VS ratio values for 30° rotation and for the best spherical compensation (named “no toric correction”) are similar.

### Influence of combined T-IOL rotation and axial shift on image simulation

**Fig 8:**
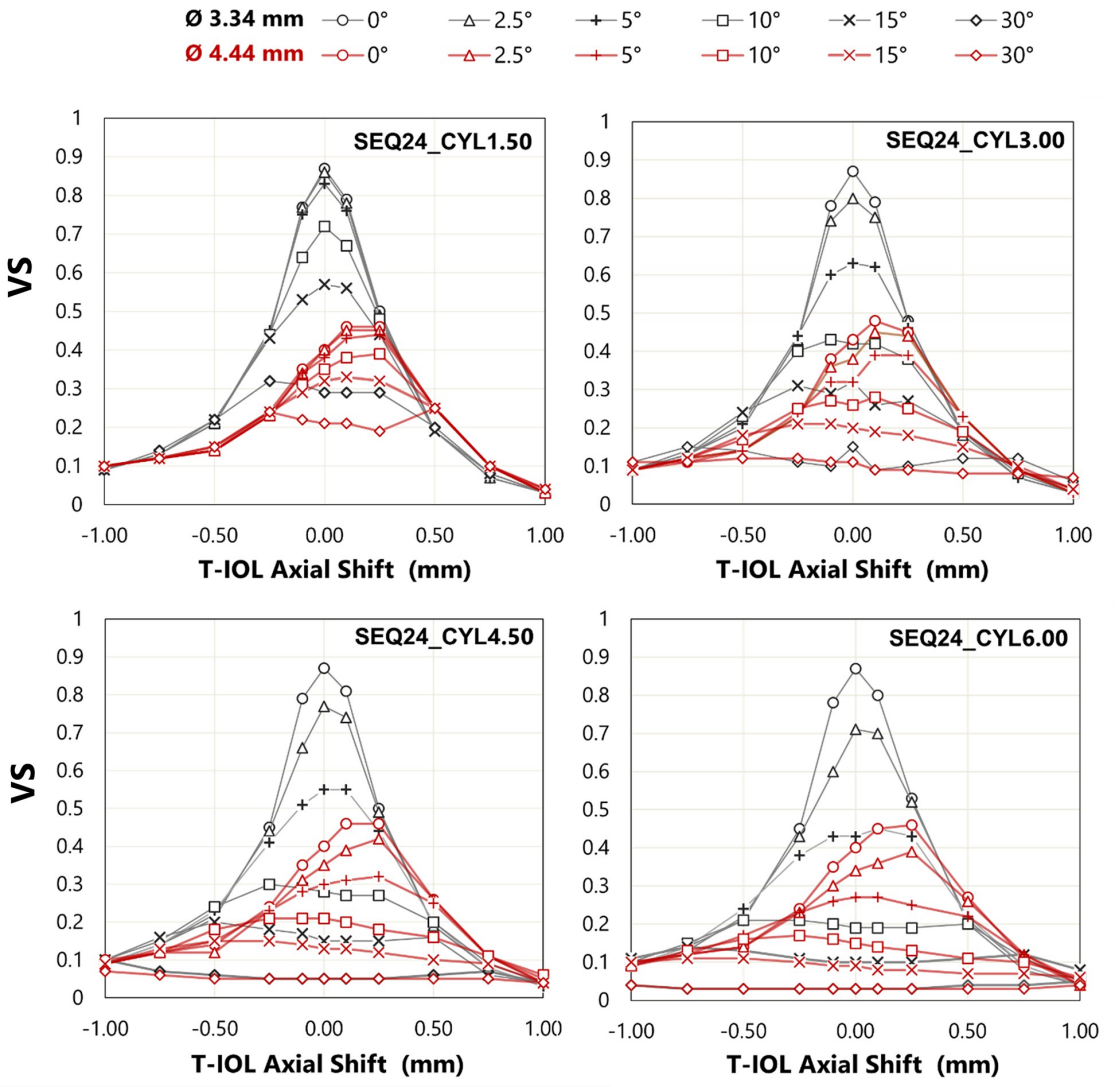
VS as a function of axial shift and rotation for a SEQ 24.00 D with four values of CYL and different entrance pupil diameters.

Axial displacement, combined with rotation, causes further decline in image quality. Fig 8 shows the VS as a function of axial shift and rotation for a SEQ with four different cylinder values and for both entrance pupil diameters. With a pupil diameter of 3.34 mm, there is a tolerance to rotation of up to 5° for axial shifts smaller than 0.25 mm, with the VS remaining above 0.6. For a 3.34 mm pupil and T-IOL with +1.50 D cylinder, the VS remains around 0.8 for rotations of 0°, 2.50° and 5° and with axial shifts ranging from -0.1 to 0.1 mm. For a +3.00 D cylinder, VS remains above 0.7 only for rotations of 0° and 2.50°, while for a +6.00 D cylinder, a VS above 0.7 is not achieved with a 2.50° rotation. For a pupil diameter of 4.44 mm, the peak of the best VS is shifted in the positive direction.

**Fig 9:**
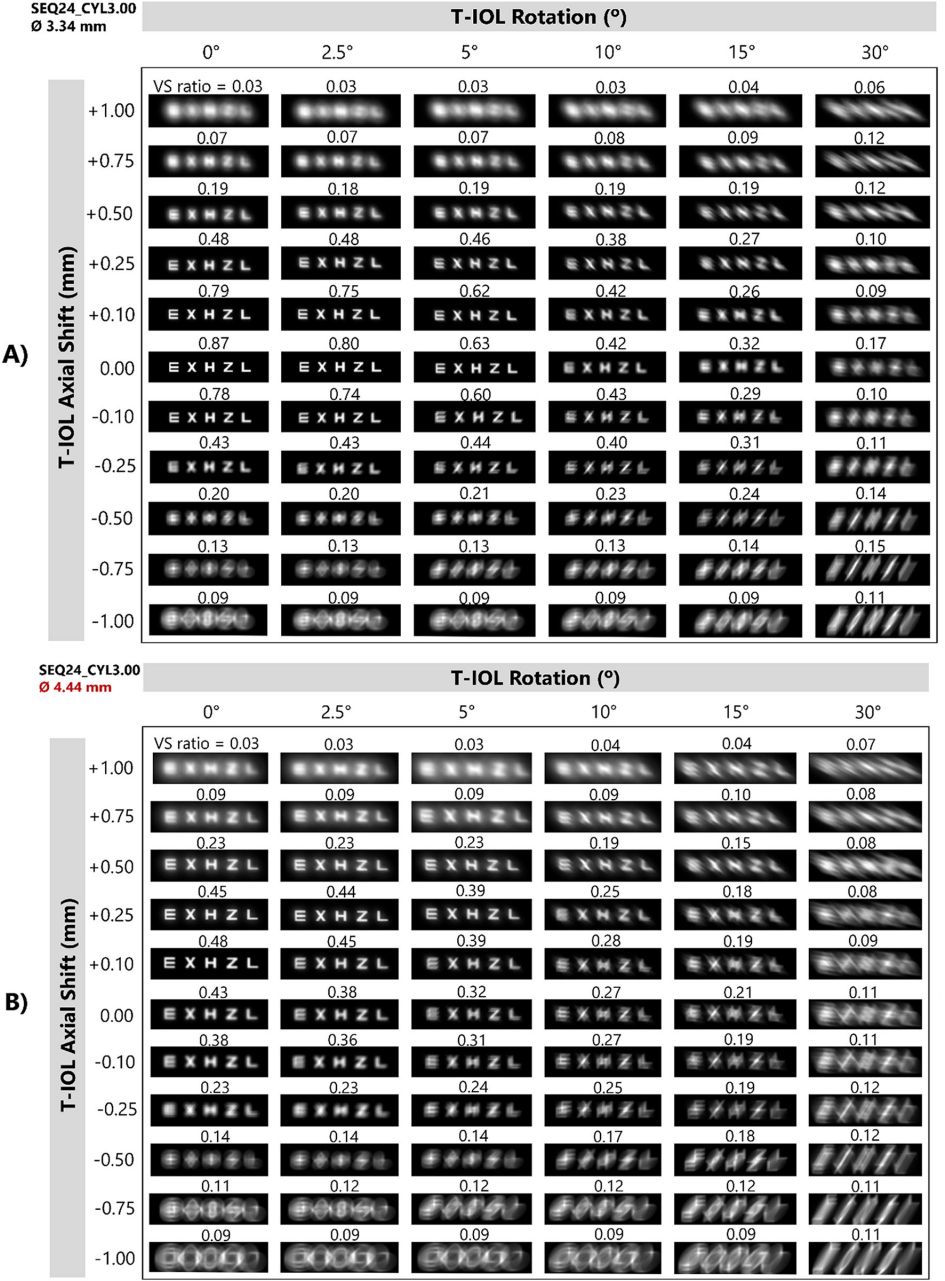
Simulated retinal images as a function of rotation for a SEQ24_CYL3.00 eye model and two entrance pupil diameters: A) 3.34 mm and B) 4.44 mm. The VS value of each simulated image is shown at the top of the image. The line of letters represents 0.2 logMAR visual acuity.

Fig 9 shows simulated retinal images resulting from the combination of rotation and axial shift for the eye model with SEQ24_CYL3.00, for both pupil diameters. In agreement with the residual spherical refraction shown at Fig 4B, the best image quality is not found at axial shift zero for the bigger pupil. As can be expected, the degradation in visual quality caused by both factors is more pronounced for the larger pupil diameter.

For the sake of brevity, only the results of the representative eye model SEQ24_CYL3.00 are shown in Fig 9. However, considering the results obtained for refractive errors in the other eye models (see for instance Fig 4), it is expected that the decrease in image quality will be greater as the SEQ and CYL values increase.

## Discussion

Several studies have reported the effect T-IOL rotation on residual refraction [20–27] but without considering the interaction with factors such as axial shift. Our investigation employed ray tracing analysis and retinal image simulations across two entrance pupil diameters.

The influence of T-IOL rotation increases with its cylinder power, but the SEQ power has only a short influence on the value of residual astigmatism (Fig 3). These findings agree with the results previously reported by Pérez-Gracia *et al*. [12].

Results were compared with the “3.33% rule” and the theoretical formula proposed by Holladay (Fig 2A) [38]. All three methods indicate a complete loss of astigmatic correction for 30° rotations for all considered systems. The results of our raytracing are close to those Holladay. Greater differences exist for the “3.33% rule” which gave the lowest results. The findings calculated with ray tracing (Fig 2B), in terms of effective loss and the residual cylinder orientation are in good agreement with the results reported by Alpins [40] who considered against-the-rule astigmatism.

The effect of axial shift of T-IOL on residual refraction was also studied. By itself, a T-IOL with a cylinder of 4.50D and a 1.00 mm axial shift had an effect of less than 0.25 D on the residual cylinder power. Additionally, any axial shift introduces an error in the M component, which shows a clear linear dependence with the SEQ (Fig 4B) across the examined range. The dependency of this relationship on the estimated IOL position and its SEQ power has been previously established [14].

We assessed the combined impact of both T-IOL rotation and axial shift on residual cylinder. Axial displacement is important when the T-IOL is not rotated. However, once a certain rotation occurs, axial displacement becomes irrelevant (Fig 5A). Fig 5B shows the change in axis with the T-IOL rotation and axial shift. Our results demonstrate how the interplay between lens rotation and axial displacement of the IOL can influence the orientation of residual astigmatism in an eye with-the-rule anterior corneal astigmatism. For small rotations of the lens, the differences in the axis of residual astigmatism are greater between different axial shifts.

VS and retinal image simulations (line of letters of +0.20 logMAR VA) were evaluated as a function of the T-IOL rotation (Figs 6 and 7) and the combined effect of T-IOL rotation and axial shift (Figs 8 and 9). The decay curve of the VS (for both pupil diameters) did not exhibit a linear pattern (Fig 6). Moreover, image quality decreased with each 5° rotation step, and was notably influenced by the CYL value. This degradation was more prominent with higher CYLs.

Regarding the VS curves exposed at Fig 6, we agree with Tognetto *et al*. [27] about the non-linear relationship between the decline in visual quality and the rotation of the T-IOL. However, our results (Fig 7) show that, after 30° of T-IOL rotation, the VS reduction is similar to “no toric correction”. The disparities between our study and Tognetto’s findings can be attributed to several factors. The most important one relates to the definition of the “no toric correction” case. In our study, in a similar way as it is done in clinical practice, the “no toric correction” case was determined with the best spherical correction in the sense of minimum RMS wavefront aberration. In Tognetto’s study, considering the appearance of the exposed “no toric correction” image it seems that image is far from the minimum RMS plane. Under this condition, Tognetto’s study is clearly underrating the visual quality which can be achieved without an astigmatic lens. Another explanatory factor for the observed differences is the use of different visual quality metrics (VS in our study and VIF in Tognetto’s study). There is no straightforward relationship to convert one into the other. Additionally, it is worth noting that VIF is a parameter dependent on a specific natural reference object (the Lena picture), whereas the VS metric is independent of the content of the reference object.

When axial displacement is combined with rotation (Fig 8), with a pupil diameter of 4.44 mm, the ’peak’ of the best VS is shifted towards positive axial shifts. This phenomenon is attributed to the emmetropia criterion that has been adopted in this work (minimum RMS wavefront error). Indeed, in the presence of SA, an RMS criterion ensures optimal image quality only under the specific pupil diameter condition employed. For an entrance pupil diameter of 4.44 mm, the VS is more tolerant to rotation than for an entrance pupil diameter of 3.34 mm. The results depicted in Fig 8 are consistent with the visual simulation images in Fig 9, which reveal an asymmetric behavior between negative and positive axial shifts in all cases, with image quality deteriorating to a greater extent for negative axial shifts.

This study had limitations. First, we did not consider aspherical T-IOLs or horizontal and vertical tilt errors. Second, chromatic aberration and asymmetric higher-order aberration were not included at the model eyes. Third, to study the influence of pupil diameter on refraction and visual quality two typical values were used, greater differences must be expected for more extremal values. Fourth, in order to save computation time, the wavefront error calculated from an ingoing ray tracing was used for both refraction and image simulation calculation. In preliminary calculations conducted by us, maximum deviations on the order of thousandths of diopters were observed in refraction calculations from outgoing ray tracing compared to those based on incoming ray tracing. Nevertheless, the validity of this approximation needs to be assessed if further study with aspherical T-IOLs, tilt errors, and/or larger entrance pupils is developed.

Future research could investigate the application of this model in clinical practice by comparing the results obtained through ray tracing with actual clinical outcomes.

## Conclusions

The study showed a better agreement between our calculations and Holladay’s formula compared to the 3.33% rule, although the differences were small.

The pupil aperture and SEQ have a minimal influence on residual astigmatism (<0.1 D), and a maximum effect of 0.25 D on residual spherical error. For our studied range, the effect of axial shift on the spherical error was independent on the sign of the displacement.

For the combined effects of rotation and axial shift, the influence of axial shift on residual astigmatism becomes insignificant for rotations exceeding 2.50°.

Concerning the VS ratio, a pupil-dependent nonlinear relationship was noted for rotation and axial shift. Contrary to findings experimental investigation, but in agreement with Holladay prediction, a rotational error of 30° is enough to reduce the visual quality of a toric lens to the highest level attainable with a spherical lens.

Considering the extensive range of cases and image quality simulations, the findings of this study will be a useful reference for vision professionals involved in refractive compensation using T-IOLs.
